# Supplementary material for: Comparative multiomics analysis of cell physiological state after culture in a basket bioreactor
Source: Sci Rep. 2022 Nov 23;12:20161. doi: 10.1038/s41598-022-24687-4 (PMC9686226; doi:10.1038/s41598-022-24687-4)
Supplement: Supplementary file 1 — Supplementary Information 1. [file 41598_2022_24687_MOESM1_ESM.zip › raw data/Transcriptomic raw data/6.Enrichment/2.KEGG/TE1vsTC1/DOWN/src/csab05205.html]

csab05205


- csab:103224900

- Down regulated genes

103224900(-1.42998371610984)

- csab:103224900

- Down regulated genes

103224900(-1.42998371610984)

- csab:103224900

- Down regulated genes

103224900(-1.42998371610984)

- csab:103220724

- Down regulated genes

103220724(-1.49708713372803)

- csab:103220724

- Down regulated genes

103220724(-1.49708713372803)

- csab:103220724

- Down regulated genes

103220724(-1.49708713372803)

- csab:103218491

- Down regulated genes

103218491(-2.95858939515048)

- csab:103220724

- Down regulated genes

103220724(-1.49708713372803)

- csab:103218213

- Down regulated genes

103218213(-1.5036177542727)

- csab:103223483

- Down regulated genes

103223483(-3.35312742084333)
- csab:103224207

- Down regulated genes

103224207(-1.32692055344416)

- csab:103220724

- Down regulated genes

103220724(-1.49708713372803)

Close
